# Special Issue: Novel Advances and Approaches in Biomedical Materials Based on Calcium Phosphates

**DOI:** 10.3390/ma12030405

**Published:** 2019-01-28

**Authors:** Michael R. Mucalo

**Affiliations:** School of Science, University of Waikato, Private Bag 3105, Hamilton 3240, New Zealand; michael.mucalo@waikato.ac.nz; Tel.: +64-7-838-4404

**Keywords:** calcium phosphates, hydroxyapatite, scaffolds, biomedical, biocompatibility, bone substitute, bone regeneration

## Abstract

Research on calcium phosphate use in the development and clinical application of biomedical materials is a diverse activity and is genuinely interdisciplinary, with much work leading to innovative solutions for improvement of health outcomes. This Special Issue aimed to summarize current advances in this area. The nine papers published cover a wide spectrum of topical areas, such as (1) remineralisation pastes for decalcified teeth, (2) use of statins to enhance bone formation, (3) how dolomitic marble and seashells can be processed into bioceramic materials, (4) relationships between the roughness of calcium phosphate surfaces and surface charge with the effect on human MRC osteogenic differentiation and maturation being investigated, (5) rheological and mechanical properties of a novel injectable bone substitute, (6) improving strength of bone cements by incorporating reinforcing chemically modified fibres, (7) using adipose stem cells to stimulate osteogenesis, osteoinduction, and angiogenesis on calcium phosphates, (8) using glow discharge treatments to remove surface contaminants from biomedical materials to enhance cell attachment and improve bone generation, and (9) a review on how classically brittle hydroxyapatite based scaffolds can be improved by making fibre-hydroxyapatite composites, with detailed analysis of ceramic crack propagation mechanisms and its prevention via fibre incorporation in the hydroxyapatite.

## 1. Introduction

The diversity of research on the development and use of calcium phosphate based biomedical materials gives rise to a thoroughly interesting and extremely interdisciplinary field of activity, from which many innovative advances can be realized for improving human health.

The importance of the field in today’s times is of unquestionable importance. Bohner [1] has commented that bone related medical conditions and bone breaks have increased due to the progressive ageing of the global population and the popularity of high risk sporting activities. In terms of the ageing aspect, Habraken et al. [2] have cited expert opinion that in the near future, 30% of all hospital admissions will be taken up by osteoporosis patients. 

In general, the main reason for pursuing calcium phosphate based bone substitutes is to reduce dependence on autologous bone, which is harvestable only in limited quantities from the iliac crest, and which necessarily subjects the patient to two surgical procedures. Donor site morbidity is also an issue. Allografts, on the other hand, are more plentiful in supply but have been said to lead to inferior healing outcomes relative to autologous bone and can potentially be a vector for disease transmission [3]. As calcium phosphate-based bone substitutes are similar in chemical composition to human bone, these have been subjected to the most research scrutiny and are relatively easy to manufacture or to source from natural sources. It is for this reason that advances in calcium phosphate based biomedical materials form the dominant subject of this Special Issue. 

Numerous reviews on state-of-the-art calcium phosphate biomaterials (and indeed biomaterials in general, constructed from other non-calcium phosphate materials) have been written. Some of these have been general in nature but others have concentrated on specific aspects or materials being actively worked upon in the field. Examples include “biphasic calcium phosphates (BCPs)” which have been described as the “gold standard of bone substitutes in bone reconstructive surgery” [4]. BCPs consist of varying mixtures of hydroxyapatite (HAp) and beta tricalcium phosphate (β-TCP), with the degree of solubility of the ceramic mixture being important. The behaviour of osteoclastic resorption was found to be influenced by the HAp/ β-TCP ratio [4]. BCPs (the term being in existence since 1986) have been successfully employed for bone reconstructive purposes in a variety of clinical situations. Other biomedical materials reviews have summarized the importance of naturally derived materials, such as animal bone (cattle bone, fish bone, etc.), in providing a feedstock source for creating calcium phosphate based materials [5]. Another active area of study is bone cements [6], which are widely used in bone reconstructive surgery due to their excellent bioresorbability, which obviates the need for xenografts or allografts that may be only semi absorbed. These materials are also useful as delivery vehicles for medications, such as antibiotics used to combat infection and biofilm production that often find an ideal milieu to proliferate on implants or bone replacement materials implanted in vivo. 

Research surrounding the effects of cationic or anionic substitutions into hydroxyapatite on its biofunctionality is also a vast activity, currently with much written the subject, as is evident in this recent review by Tite et al. [7], though this only concentrates on cationic substitutions. The doping of cations or anions into hydroxyapatite has been shown to have a number of beneficial effects, such as increasing biological activity in terms of cytocompatibility, cell viability and proliferation, adhesion, haemocompatibility, antimicrobial or antifungal factors, among other effects. However, the field is crowded with many different studies, and some degree of comparison between studies to ascertain the best procedures to follow is needed to drill down into what exactly is causing the beneficial effects of a cation or an anion substituted HAp lattice.

Ceramic composites are also an active area of study [8], with the intent being to more closely mimic bone, which can be described as a living composite of bone and collagen [9]. Synthetic HAp on its own is too brittle and clearly unsuitable for load-bearing applications due to the risk of cracking and failure. Composite scaffold production has the aim of producing materials with superior mechanical properties.

Other work has shown that processing conditions and controlling aspects as fundamental as the pH of the solution in which the hydroxyapatite is generated can influence biological response [10]. This can alter surface properties, such as zeta potential, which can, among other factors, dictate the success of cell interactions with the implanted biomedical material.

## 2. Synopsis of Papers Contributed to the Special Issue

In discussing some of the main areas of biomedical materials research involving calcium phosphate-based materials above, this Special Issue aimed to attract papers describing the latest advances, and successfully led to nine papers being accepted for publication that provide a very interesting snapshot into some very topical areas of biomedical research involving calcium phosphate-based materials. A brief summary of these is provided below and interested readers are invited to seek further information by referring to the actual articles embodied in this Special Issue.

The paper by Bakry et al. [11] contains a dental materials research theme. Orthodontic patients can frequently suffer from decalcification of enamel surfaces adjacent to their orthodontic devices due to problems with oral hygiene. These so-called white spot lesions are difficult to treat and can lead to dental caries, which can become unsightly. This paper examined the use of a novel fluoride-containing bioactive glass paste known as BiominF for remineralization of white spot lesions associated with orthodontic treatment. The study used transverse micro radiography and scanning electron microscopy/energy dispersive spectroscopy (SEM/EDS) techniques to observe remineralization events on enamel demineralized surfaces.

In the paper by Rakhmatia et al. [12], an investigation into how statins can enhance bone formation was carried out. This was executed by evaluating how readily apatite blocks made via a dissolution-reprecipitation reaction, involving preset gypsum in the presence and absence of statins, could increase bone formation in a socket healing scenario after tooth extraction. Four week old male Wistar rats were used as the model to test this hypothesis. Carbonate apatite containing statins were found to exhibit very favourable results for enhancing bone mineral density in the vertical plane showing it could not only allow but promote bone healing in socket healing. This result could, in the future, be a strategy for the preservation of alveolar bone after tooth extraction.

The research reported in this paper by Mitran et al. [13] followed the theme of deriving biomedical materials from naturally sourced feedstocks. Bioceramic materials were obtained from processing of dolomitic marble and indigenously sourced *Mytilus galloprovincialis* seashells via the decomposition of the calcium carbonate minerals contained within the natural powders into CaO, hydrolysis to form Ca(OH)_2_, and then mixing with phosphoric acid to form pellets. These were found to contain a biphasic composition involving largely hydroxyapatite and brushite. These were then evaluated for their bone regenerative capabilities by investigating the interaction of pre-osteoblastic cells, namely MC3T3-E1 cells, with the materials in terms of their adhesion properties, morphological characteristics, viability, proliferation, and differentiation. The *Mytilus galloprovincialis* seashell-derived material was found, in particular, to efficaciously induce cell differentiation in these studies on a par with the performance of the reference hydroxyapatite materials used.

Khlusov et al.’s paper [14] detailed the very interesting area of surface electrical charge and topography of biomedical materials and their influence on cell responses to these materials.

In this in vitro study, the authors investigated the relationship between the roughness of calcium phosphate surfaces and the surface electrical charge, and its possible role in affecting human MSC (mesenchymal stem cells) osteogenic differentiation and maturation. The behaviour of these in vitro was estimated by human adipose-derived MSCs or prenatal stromal cells from the human lung. A microarc calcium phosphate coating was prepared on a titanium substrate and characterized, although was found to be amorphous to X-ray in its as-prepared form, hence necessitating its annealing at 1073 K to crystallize the phases present to enable identification. These were revealed to be a mixture of calcium titanium phosphates, calcium/titanium pyrophosphates, and anatase. The authors then used human adult adipose derived MSCs or human lung derived prenatal stromal cells (abbreviated in the paper as “HLPSCs”) and cultured them on the calcium phosphate surfaces to estimate MSC behaviour. It was demonstrated that roughness, non-uniform charge polarity, and the electrical potential of the microarc coatings affected the osteogenic differentiation and maturation of the cells in vitro. Electrical potential on the surface of the coatings increased with increasing roughness at the surface (at microscale). It was found that nanoscale surface features influenced the sign of the electrical potential with location of negative electrical charges mostly existing in micro and nanosockets of the coating surface. Positive charges mostly existed at nanorelief peaks. In the sockets, HLPSCs were located and expressed osteoblastic markers osteocalcin and alkaline phosphatase. The topography of the coating promoted the osteoblast phenotype of HLPSCs. Overall, it was thought that the negative sign existing at the calcium phosphate surfaces and its magnitude at various locations corresponding to micro and nanosockets could be factors that stimulate osteoblastic differentiation and maturation of human stromal cells.

Rheological and mechanical properties of a novel injectable bone substitute are the theme of the paper by Oğuz et al. [15]. This was accomplished by mixing a bioceramic powder in a solution of polymers consisting of methylcellulose, gelatin, and citric acid. Methylcellulose was used because of its thermoresponsive and biocompatibility properties. Added gelatin and citric acid served to adjust rheological properties in the injectable bone substitute. The bioceramic powder component comprised tetracalcium phosphate, hydroxyapatite, brushite, and calcium sulfate dihydrate, which was added in proportions up to 50 wt%. The so-prepared injectable bone substitute had a “chewing gum” consistency. Characterisation was carried out on the materials in terms of chemical structure, rheological, and mechanical properties. Hardening of the injectable material was confirmed at physiological temperature. In addition, in vitro degradation studies were carried out. These indicated a lower rate of degradation as the wt% of bioceramic powder increased, with a concomitant improvement in mechanical properties. Overall, the injectable bone substitute material was judged a promising candidate for treatment of bone defects in non-load bearing applications.

In yet another bone-cement-themed paper, this time by Boehm et al. [16], the authors considered solutions to the long term and well known problem of the brittleness of calcium phosphate cements, which leads to low fracture toughness. This, of course, limits them to non-load bearing applications in clinical practice. Carbon fibre reinforcement is known to improve fracture resistance, but at the same time compromise strength of the composite. It was found that chemically modifying the fibre surfaces and using these fibres to reinforce the calcium phosphate cements resulted in a modification of the fibre matrix interface and the fracture behaviour. In taking this approach, the authors were able to demonstrate enhanced mechanical properties as regards bending strength and work of fracture to a strain of 5%. They found that using fibre reinforcement did not affect the cell proliferation and activity of MG63 (human osteoblast-like) cells that were used as biocompatibility markers. The conclusion was that the use of chemically activated C-fibres to reinforce calcium phosphate cements was a promising method to achieve better mechanical properties in these bone cement replacement materials to the extent that they might be of use in load bearing applications.

Farré-Guasch et al.’s paper [17] tries to address the classical issue encountered with xenograft type materials in regard to their lack of osteoinductive and angiogenic properties. This paper concentrated on the potential of adding adipose stem cells (ASC) to calcium phosphate based scaffolds in order to stimulate osteogenesis, osteoinduction, and angiogenesis in them. The bone substitute (“calcium phosphate carrier”) materials used for seeding the (autologous) ASC-containing stromal vascular fraction from ten patients were (pure) β-tricalcium phosphate and biphasic calcium phosphate. These were used in a human maxillary sinus floor elevation model in a one-step surgical procedure. After six months of implantation, biopsies collected quantified blood vessel formation and bone percentages. Bone percentages were found to correlate with blood vessel formation and were higher in study subjects compared to control biopsies in the cranial area. This was particularly so with the β-tricalcium phosphate blocks. The study showed the pro-angiogenic effect of using the stromal vascular fraction to seed the calcium phosphate scaffolds.

The paper by Salamanca et al. [18] is concerned with surface treatments for the purpose of removing contaminants on biomedical material surfaces. To achieve this, glow discharge plasma treatments on calcium phosphate biomaterial surfaces were carried out. The point of removing contaminants was in order to facilitate cell attachment and enhance bone regeneration. These contaminants can arise from inorganic grit blasting media (which can be Si or Al-based oxides) residues or layers arising from processing fluids, such as etchants or cleaning solvents, or substances that remain after sterilization procedures, like autoclaving or the use of ethylene oxide. In addition, most surfaces exposed to ambient air can pick up organic contamination (often known as “adventitious hydrocarbons” in surface science [19]). In this study, calcium phosphate composite materials consisting of hydroxyapatite and β-tricalcium phosphate were treated with argon glow discharge plasma for 15 min at room temperature and were then subjected to scanning electron microscopy/energy dispersive spectroscopic (SEM/EDS) analysis. These showed that low levels of metal ion impurities at the surface had been removed by the glow discharge process. Other biological tests, such as cell viability, morphology, and an alkaline phosphatase assay, were also conducted on untreated and glow discharge treated calcium phosphate surfaces and compared to controls. Improved cell proliferation, increased alkaline phosphatase activity, and enhanced differentiation into osteoblast like cells were observed after 5 days on the argon glow discharge treated substrates. No chemical modification was noted to occur to the bulk calcium phosphate materials. Thus, the improvement was directly ascribed to the argon glow discharge treatment of the surface, though it was recommended that further testing be conducted in the in vivo models to further prove its efficacy.

The review by Siddiqui et al. [20] discusses the use of hydroxyapatite as a bone-substituting and osteoconductive scaffold, but also comments on the classical problem of implants made from (pure) hydroxyapatite having poor mechanical properties when placed under load. This limits their usage to non-load bearing applications. In order to make hydroxyapatite more suitable for load bearing applications or to achieve better mechanical properties, it is necessary to create composites of hydroxyapatite with other materials, whilst maintaining the biocompatibility of the resultant material. The review then moves on to discuss the strengthening and toughening mechanisms which are crucial to understand if the aim is to produce new and optimized materials from the compositing approach. The main engineering conundrum when designing a material that is both strong and tough is that often an increase in strength can be coupled with a decrease in toughness, and vice versa. A detailed discussion is provided on fracture mechanics, which constitutes a valuable tool to analyze the conditions under which a crack may propagate and eventually lead to failure. Further discussion concentrates on how crack propagation can be prevented, which in brittle ceramic materials is known as a process of extrinsic toughening.

## 3. Concluding Remarks

As is obvious, a diverse range of topics has been covered in this Special Issue, although it is clear that there is an underlying emphasis on how the biological processes, such as cell proliferation and others, can be facilitated or enhanced on the prepared calcium phosphate biomaterials when implanted. This remains of critical importance when engaging in this research area. It is hoped that the readers will find this collection of research of interesting reading for advancing understanding in calcium phosphate-based biomaterials.
